# Microbiota-gut-brain axis and ketogenic diet: how close are we to tackling epilepsy?

**DOI:** 10.20517/mrr.2023.24

**Published:** 2023-08-29

**Authors:** Mariachiara Mengoli, Gabriele Conti, Marco Fabbrini, Marco Candela, Patrizia Brigidi, Silvia Turroni, Monica Barone

**Affiliations:** ^1^Microbiomics Unit, Department of Medical and Surgical Sciences, University of Bologna, Bologna 40138, Italy.; ^2^Unit of Microbiome Science and Biotechnology, Department of Pharmacy and Biotechnology, University of Bologna, Bologna 40126, Italy.

**Keywords:** Ketogenic diet, gut microbiome, epilepsy, dietary intervention, microbiome-based therapeutics, microbiota-gut-brain axis

## Abstract

The microbiota-gut-brain axis refers to the intricate bidirectional communication between commensal microorganisms residing in the digestive tract and the central nervous system, along neuroendocrine, metabolic, immune, and inflammatory pathways. This axis has been suggested to play a role in several neurological disorders, such as Parkinson’s disease, Alzheimer’s disease, multiple sclerosis, and epilepsy, paving the way for microbiome-based intervention strategies for the mitigation and treatment of symptoms. Epilepsy is a multifaceted neurological condition affecting more than 50 million individuals worldwide, 30% of whom do not respond to conventional pharmacological therapies. Among the first-hand microbiota modulation strategies, nutritional interventions represent an easily applicable option in both clinical and home settings. In this narrative review, we summarize the mechanisms underlying the microbiota-gut-brain axis involvement in epilepsy, discuss the impact of antiepileptic drugs on the gut microbiome, and then the impact of a particular dietary pattern, the ketogenic diet, on the microbiota-gut-brain axis in epileptic patients. The investigation of the microbiota response to non-pharmacological therapies is an ever-expanding field with the potential to allow the design of increasingly accessible and successful intervention strategies.

## INTRODUCTION

**Figure 1:**
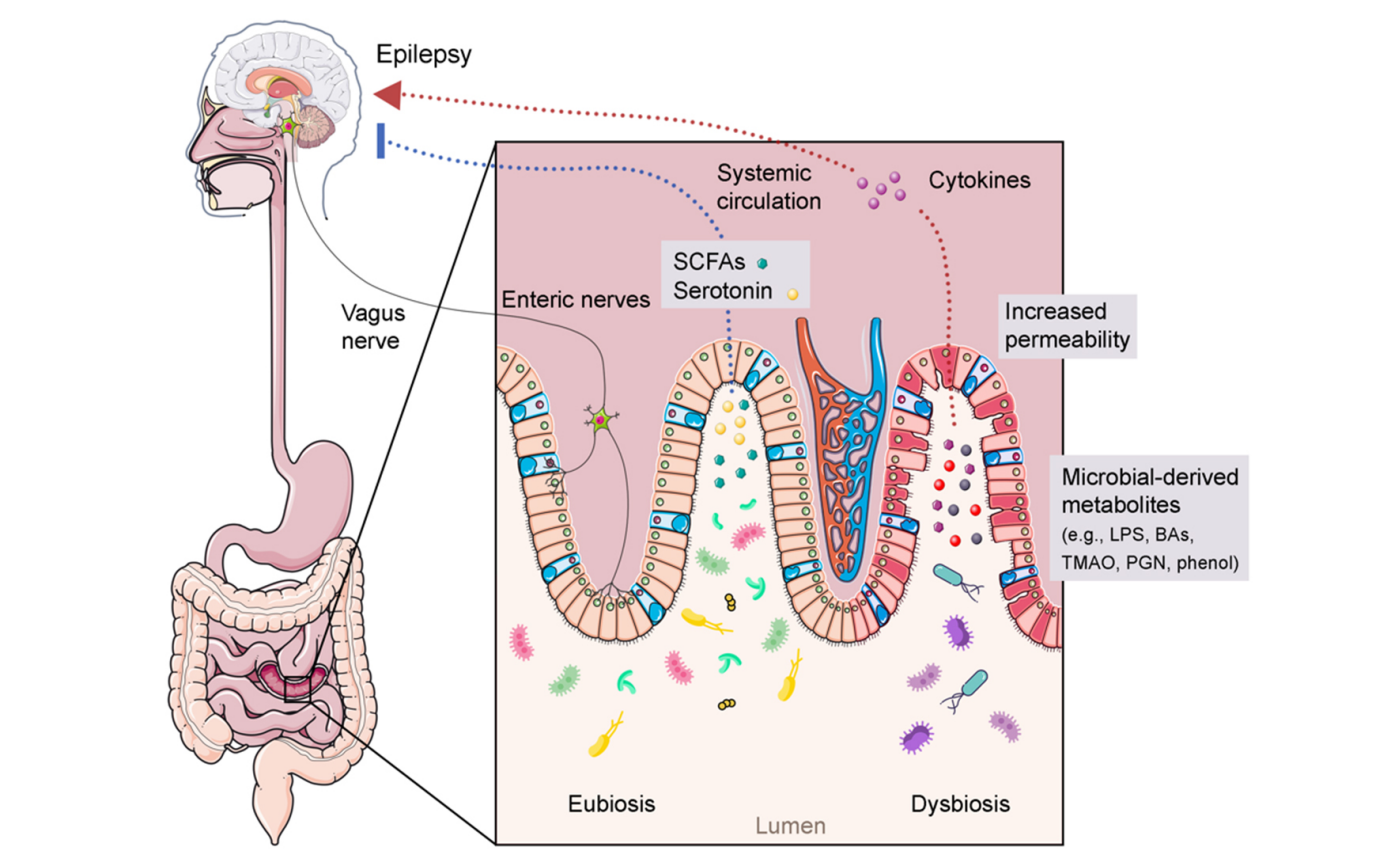
The microbiota–gut–brain axis in epilepsy. The gut microbiome could affect epilepsy through several mechanisms. For example, an eubiotic microbiota produces metabolites, such as SCFAs and serotonin, which are known to have a possible inhibitory activity on epilepsy occurrence. On the other hand, a dysbiotic layout could be associated with increased gastrointestinal permeability and the production of epilepsy-promoting metabolites, such as proinflammatory factors, which could disrupt the host GABA/glutamate ratio inducing epilepsy events. The interaction between the gut microbiota and the enteric/vagus nervous system, and HPA axis are also potentially involved in the manifestation of epileptic events. BAs: Bile acids; GABA: γ-aminobutyric acid; HPA: hypothalamic-pituitary-adrenal; LPS: lipopolysaccharide; PGN: peptidoglycan; SCFAs: short-chain fatty acids; TMAO: trimethylamine N-oxide.

The bidirectional connection between microbiota, gut, and brain has been extensively studied over the past two decades. In particular, studies focusing on the microbiota-gut-brain axis (MGB) have been highlighted as crucial for a paradigm shift in the field of neuroscience^[1-4]^. The MGB axis involves numerous physiological pathways at the neural, endocrine, metabolic, and immune system levels [Figure 1]^[5]^. Among these, the enteric nervous system, an important component of the nervous system, innervates the entire gastrointestinal tract (GIT) and has the potential to regulate many gastrointestinal physiological functions through central nervous system (CNS)-independent actions^[6]^. In light of this, the MGB axis may represent an interesting link between neurological pathologies, mental disorders, and gut health^[7]^. Indeed, the MGB axis has been proposed as an innovative avenue for investigation in neuroscience^[8]^.

The gut microbiota (GM) plays a key role in supporting host physiology, contributing to CNS development, and promoting processes such as neurogenesis, microglial maturation, and myelination. GM may also influence the pathogenesis and progression of several childhood and adult neurological disorders, including Parkinson’s and Alzheimer’s diseases, epilepsy, autism spectrum disorder and multiple sclerosis^[5,9-12]^. On the other hand, it has been suggested that neurotransmitters may act at the level of the GIT innate immune system, control and regulate blood flow, intestinal motility, nutrient absorption, and, not least, GM. In support of this, in some pathological conditions, such as Parkinson’s disease (PD), neurotransmitter levels are often imbalanced and lead to different symptoms at the GIT level^[13]^. Furthermore, some GM components can produce or modulate the levels of neuroactive metabolites, such as short-chain fatty acids (SCFAs), but also dopamine, serotonin (or 5-hydroxytryptamine-5-HT), and γ-aminobutyric acid (GABA)^[14]^, as will be discussed in the following paragraphs.

Epilepsy is a neurological disease that affects 50 million people worldwide and is characterized by the predisposition of the brain to generate seizures^[15]^. This neurological condition can be classified as focal, generalized, combined, or focal-generalized, depending on the type of seizures. The association with a history of previous lesions at the level of the nervous system is also taken into account. In this case, the epilepsy is called post-traumatic, i.e*.*, occurring after a traumatic brain injury, and is a major cause of permanent disability and death^[16]^. The inflammatory state associated with other diseases is also a discriminator of the type of epilepsy. For example, patients with Crohn’s disease have an epilepsy incidence of 3.5%-5.5%, while general neurological manifestations occur in 33%-67%.

The most common antiepileptic treatments are drug-based, but in > 30% of patients, drug therapy is not sufficient to control seizures, a phenomenon known as refractory epilepsy^[11]^. It is therefore of the utmost importance to develop more effective protocols for the treatment of epilepsy. Among the non-pharmacological approaches proposed for drug-resistant epileptic patients unsuitable for surgery, the ketogenic diet (KD) has long been used with positive outcomes^[15]^. This clinical evidence supports a close interconnection between the gut, its microbial inhabitants, and epilepsy. In parallel, the diffusion of multi-omics approaches has paved the way for greater awareness of the role played by GM in epilepsy, especially in subjects refractory to conventional therapies^[5,17-20]^. The next step undoubtedly concerns the identification and study of the biological mechanisms underlying the role of the MGB axis in epilepsy, with the implementation of new study models and the design of therapeutic interventions aimed at modulating the GM to improve clinical outcomes. Considering the beneficial impact on host health of the recovery of a state of intestinal eubiosis, a first-hand approach based on personalized diets such as the promising KD could become the future standard treatment of refractory epilepsy. Herein, we first review the latest knowledge on the mechanisms underlying MGB axis involvement in different types of epilepsy (i.e*.*, refractory, temporal lobe, idiopathic focal, and drug-resistant), then discuss the impact of antiepileptic drugs and KD on GM, and finally summarize the impact of tailored dietary interventions as increasingly accessible non-pharmacological therapies to tackle epilepsy. Critical issues in the field are also discussed.

## MICROBIOTA-GUT-BRAIN AXIS AND CORRELATIONS WITH EPILEPSY

### Role of inflammation and immune responses in the central nervous system

Neuroinflammation and neuroimmunity have been linked to the pathogenesis of epilepsy^[20]^, and more recently, accumulating evidence has brought to the fore the potential involvement of inflammatory and immune pathways in the MGB axis^[21]^. The onset of epilepsy is, in fact, triggered by the inflammatory state of the main inflammatory cells of the CNS, i.e*.*, microglia and astrocytes^[22,23]^. Astrocytes, the most abundant cells in the brain, perform a wide range of physiological functions, including participation in immune responses, neurotransmitter recycling, and regulation of the integrity of the blood-brain barrier (BBB)^[24]^. On the other hand, microglia, which are composed of CNS resident macrophages, are responsible for the innate immune response in the brain^[25]^. Among microglial cells, the largest and least ramified cells with an amoeboid morphology are capable of triggering inflammatory processes, regulating neuronal activity and clearance of phagocytic neurons, and promoting the onset of chronic seizures^[26]^. Indeed, microglial and astrocyte functions are involved in the pathogenesis of epilepsy through the excessive release of cytokines^[22]^. The two cell populations interact synergistically, with microglia modulating the phenotype and function of astrocytes^[27]^, which in turn regulate the inflammatory response^[28,29]^. The dynamic interconnection between astrocytes and microglia results in a stimulation of proinflammatory cytokine production and BBB permeability, with the consequent triggering of the infiltration process of peripheral blood immune cells and cytokines into the CNS, promoting the establishment and sustenance of a chronic neuroinflammatory state^[30]^. On the other hand, the release of inflammatory cytokines and chemokines by astrocytes has been linked to an enhancement of microglial activities, resulting in a stimulating action of numerous physiological processes, including migration, phagocytosis of apoptotic cells, and synaptic pruning^[31]^. Studies in animal models have highlighted vascular endothelial growth factor β (VEGF-β) and transforming growth factor-α (TGF-α) as modulators, respectively, of the pro- and anti-inflammatory astrocyte response mediated by pathogen-induced microglial activation^[28]^. Inflammation and immune responses in the brain may also be secondary to the invasion of peripheral immune cells, including those derived from the GIT, or to the translocation of GM-related components, as discussed in the next paragraph.

### Role of inflammation and immune responses in the gastrointestinal tract

From the GIT standpoint, 70%-80% of the human body’s total immune cells are encased within the lymphoid tissue of the intestinal mucosa^[32]^. Moreover, it has been demonstrated that the GM exerts an influence on immune cells. In germ-free (GF) mice, numerous immune abnormalities have been found, which are reflected in a reduction in the population of T and B cells together with a consequent decrease in the production of cytokines^[33]^. The GM also represents one of the most relevant factors for the maturation of microglial cells, as well as the activation of astrocytes^[34]^, in an age- and sex-dependent manner^[35]^. Innate immunity, adaptive immunity, and inflammatory mechanisms underlying epilepsy development can thus be regulated by the GM. GM members and microbially derived metabolic products are compartmentalized due to the crucial synergistic action of the intestinal mucosal barrier and BBB, preventing entry into the brain. When these barriers are broken down, intestinal permeability increases – as in the case of leaky gut syndrome - and as a result, the translocation of microorganisms, metabolites and other molecules into the bloodstream occurs^[36]^. The permeability of the intestinal mucosa may be affected even under stress conditions, for example, allowing lipopolysaccharides present in the intestinal lumen to enter the circulation and stimulate Toll-like receptors, with consequent production of inflammatory cytokines capable of altering the BBB, increasing its permeability, and ultimately damaging the brain^[37,38]^. Similarly, in a scenario of intestinal inflammation, the release of bacterial mediators into the systemic circulation can trigger a response mediated by the activation of peripheral immune cells, with alteration of the BBB and transport rates, which can result in pathological conditions such as the leaky brain^[39]^.

From the perspective of the MGB axis, the neuroimmune response is also affected by the GM. Gut microorganisms are in fact capable of bioconverting food-derived tryptophan into aryl hydrocarbon receptor agonists, driving microglial activation and TGF-α and VEGF-β expression, thereby regulating the activity of astrocytes^[28,29]^. GM composition and diversity are critical for microglial and CNS function, as suggested by numerous studies conducted on GF animals^[40,41]^. In particular, GF animals and antibiotic-treated animals showed impaired microglial morphology as well as defects in its maturation, activation, and differentiation, resulting in an inadequate immune response against various pathogens, a function restored only following recolonization of the GIT by commensal microorganisms^[40]^. At the CNS level, peripheral immune cells such as T cells and monocytes that invade brain tissue have been found to be involved in epilepsy. Noteworthy, CNS invasion can also involve of macrophages resulting from monocyte differentiation. In the cerebral district, infiltrated macrophages differentiate into microglia-like cells, contributing to epilepsy^[42]^. This process most likely sees the involvement of the GIT, as it represents the organ with the largest population of immune cells. However, further investigations are needed to identify and characterize the precise mechanisms underlying this involvement.

With specific regard to the GM-immune system interactions, epilepsy can be induced through GM-mediated innate immune responses. In GF mice, reduced expression of claudin-5 and occluding proteins in the endothelium causes a progressive increase in BBB permeability over the course of life^[43]^. The reduced production of claudin can also occur in response to the establishment of dysbiotic GM layouts, increasing the permeability of the intestinal epithelium, undermining its physiological barrier function, and favoring the translocation of microorganisms, metabolites, and toxins from the intestinal lumen towards other districts^[44]^. Dysbiotic GM states also often result in decreased production of bacterial fermentation endpoints such as SCFAs, which may promote neuroinflammation by increasing BBB permeability^[45]^. When the barrier effect fails in both intestinal and cerebral compartments, immune cells and GM-derived factors can enter the brain, ultimately inducing seizures. Among bacterial-derived molecules, peptidoglycan (PGN) translocated outside the GIT to the CNS has been suggested to be responsible for promoting brain leakage, triggering chronic inflammatory responses, and contributing to the onset of epilepsy. In particular, PGN has been detected in cerebral microglia and identified as a driver in the onset of chronic encephalitis^[46]^. PGN has also been related to the occurrence of sclerosis-related temporal lobe epilepsy^[47]^. GM-triggered adaptive immune responses may also play a role in the generation of epilepsy. Immune cells can be stimulated by GM to produce cytokines that migrate to the brain district first through the intestinal mucosa and then the BBB, engaging brain immune cells to contribute to the adaptive immune response. Among the key components of adaptive immunity, T helper 17 (Th17) cells are responsible for the production of the proinflammatory cytokine interleukin-17 (IL-17)^[48]^. Specific bacterial phyla, including Bacteroidetes, have been linked to modulation of Th17 proinflammatory activity^[48,49]^. Accumulating evidence suggests not only the involvement of IL-17 in epilepsy, but also its correlation with seizure frequency and severity. Supporting this correlation, increased levels of this proinflammatory cytokine have been observed in the cerebrospinal fluid and peripheral blood of patients with epilepsy compared with healthy subjects^[50-53]^. Other microbially derived metabolites, such as SCFAs, may play a role in the synthesis and secretion of immunoglobulins, affecting B lymphocyte differentiation^[54,55]^. In GF animals, greater susceptibility to disease is observed, as the total absence of commensal microorganisms causes an upregulation of immunoglobulin E (IgE) at the expense of immunoglobulin A (IgA) and immunoglobulin G (IgG), which are instead downregulated^[56,57]^.

### Autonomous nervous system

Among the pathways of information exchange and communication between the gut and the brain, one of the most important is undoubtedly through the autonomic nerve fibers. Being involved in maintaining bodily homeostasis as a component of the parasympathetic branch of the autonomic nervous system, the vagus nerve tonically transmits information from the viscera to the brain and vice versa. From the perspective of our microbial counterpart, the fastest and most direct way for the GM to affect the brain is to hijack vagus nerve signaling. In a landmark study, Goehler *et al.* demonstrated that oral inoculation of *Campylobacter jejuni* in a murine model was linked to increased expression of the proto-oncogene c-fos in the sensory ganglia and primary sensory nucleus of the vagus nerve in the brainstem, suggesting that intestinal stimulation can affect brain activity via the autonomic nervous system^[58]^. Vagus nerve stimulation, developed for the first time in 1988^[59]^, is currently one of the routine therapies proposed for epilepsy. Chronic stimulation of the left vagal nerve using a subcutaneous stimulator connected to electrodes placed in the cervical region is a low-risk but expensive procedure that reduces seizure frequency in about 50% of patients with otherwise untreatable severe epilepsy^[60]^. The vagus nerve winds through all layers of the GIT but does not interact directly with GM due to its absence at the epithelial level^[61]^. However, it has been observed that electrical stimulation of vagal afferent fibers is potentially capable of influencing the levels of neurotransmitters such as 5-HT, GABA, and glutamate in the brain, thus legitimizing its application in epilepsy^[62]^. On the other hand, there are no studies reporting the effects of vagus nerve stimulation on GM in animal models or in patients with epilepsy. Similarly, although promising, literature data on the effects of vagotomy are still scarce and at a preclinical level. In a first study, Krahl *et al.* investigated the seizure-suppressing effects of systemic epinephrine in rats. Interestingly, vagotomy hindered this effect, demonstrating that epinephrine-induced seizure suppression was mediated by subdiaphragmatic vagal afferents^[63]^. In a second study, Bhandare *et al.* investigated the impact of vagotomy in male Sprague-Dawley rats, monitoring cardiovascular autonomic dysfunction and highlighting that seizure-induced sympathoexcitation was caused by activation of glutamatergic receptors in the rostral ventrolateral medulla, which also caused proarrhythmogenic changes mediated by pituitary adenylate cyclase-activating polypeptide and microglia^[64]^. Taken together, these results suggest that the peripheral vagus nerve pathway may be exploited to develop new antiseizure treatments. However, as discussed above, no *in vivo* experiments have yet been conducted on the ablation of neuronal fibers afferent to the vagus nerve. Furthermore, no studies have investigated the potential impact of these vagus nerve-related approaches on the GM counterpart and whether and how the latter might contribute to the success of such vagus nerve-related therapies.

Among other cellular mechanisms through which GM communicates with the brain, enteroendocrine cells (EECs), via their numerous receptors, play a pivotal role in the detection of signals released by intestinal microorganisms at the luminal level. However, EECs do not communicate with the cranial nerve solely and exclusively by hormone-based signaling^[65]^. The study of Kaelberer *et al.* has in fact highlighted that a type of EECs, called neuropod cells, are able to transduce luminal signals and enter into synapses with vagal neurons, in such a way as to connect the intestinal lumen to the brain using glutamate as a neurotransmitter^[65]^. These findings further highlight the possibility of mitigating and/or treating some neurological diseases, such as epilepsy, through intervention strategies based on GM modulation.

### Microbial metabolites in enteroendocrine signaling: short-chain fatty acids and neurotransmitters

SCFAs, such as acetate, propionate, and butyrate, are the endpoint of the fermentation processes of insoluble dietary fibers by various commensals present in the GIT^[66]^. As mentioned above, SCFAs play a fundamental role in processes closely related to epilepsy, such as microglial and nervous system maturation, BBB permeability, and modulation of stress responses through both direct and indirect pathways^[67]^. The effects and protective mechanisms of SCFAs in epilepsy have been investigated thanks to studies conducted on pentylenetetrazol-induced kindling epileptic mouse models^[68-70]^. In particular, the anticonvulsant effect linked to butyrate depletion has been highlighted^[71]^, along with the butyrate potential to relieve intestinal inflammation and oxidative stress^[67]^. The protective effect of butyrate can increase the seizure threshold and reduce seizure intensity, while protecting brain tissue and neuronal apoptosis and improving mitochondrial dysfunction^[69]^. Propionate has also been shown to be useful in alleviating seizure intensity by reducing mitochondrial damage, hippocampal apoptosis, and neurological deficits^[70]^. The reduction in SCFA levels has been observed in several models of epilepsy, allowing the identification of their protective potential on epilepsy and the potential underlying mechanisms.

Neurotransmitter imbalance is frequently observed in patients with epilepsy and has emerged as one of the most frequent pathophysiological alterations in this neurological disease. Specifically, derangements of GABA and 5-HT, related to hypoactivity, as well as glutamate, dopamine, and norepinephrine, related to hyperactivity, have been found in epileptic epidemics^[72]^. Several studies have highlighted the production of neurotransmitters and/or their precursors also in the GIT as a result of microbial metabolism or GM-dependent stimulation of gastrointestinal cells. The researchers highlighted that the pool of neurotransmitters in the intestine can vary according to the compositional layout of the GM. In particular, 5-HT is closely related to several bacterial species belonging to the genera *Enterococcus*, *Escherichia*, and *Streptococcus*. GABA is associated with *Bifidobacterium* and *Lactobacillus*, while norepinephrine and dopamine are attributable to the presence of *Escherichia* and *Bacillus*. Translocation of neurotransmittersfrom GM metabolism across the intestinal mucosa is very frequent. On the other hand, BBB remains impervious to most of them, allowing itself to be crossed only by GABA^[73]^. In epilepsy or cases of hippocampal injury, an important role has been attributed to GABA produced by GM. In particular, an alteration of the GABA/glutamate balance capable of inducing convulsions has been found. By analyzing the GM composition in epileptic patients, a greater representation of the genera *Coprococcus*, *Ruminoccus* and *Turicibacter* was found, as well as a positive correlation between the relative abundances of these microorganisms and the levels of both glutamate and glutamine^[74]^. In addition to the production of neurotransmitters, GM is able to influence the glutamine-glutamate-GABA cycle and ultimately mediate the expression of GABA and N-methyl-D-aspartate receptors in the brain, specifically in the hippocampus, amygdala, and locus coeruleus^[67]^. Studies conducted on murine models with seizures electrically induced have shown protective anticonvulsant effects following intestinal colonization by *Parabacteroides* and *Akkermansia muciniphila*, reflected in modulation of amino acid levels in the intestinal lumen and serum that affect hippocampal levels of neurotransmitters associated with seizures (i.e*.*, GABA and glutamate)^[18,75]^. As for 5-HT, enterochromaffin cells in the gut produce about 90% of it^[76]^, and a 5-HT deficiency has been observed in patients with temporal lobe epilepsy. It is interesting to note that selective serotonin reuptake inhibitor drugs, taken individually or in combination, are able to increase 5-HT levels, resulting in an improvement and control of seizures in patients with epilepsy^[77]^. On the contrary, the reserpine-induced decrease in 5-HT appears to increase susceptibility to minimal electroshock-induced seizures in rats^[78]^. The biosynthesis of 5-HT in the intestine can also be promoted by some GM components (e.g., spore-forming clostridia). Studies conducted on GF mouse models have in fact highlighted an alteration in the levels of 5-HT, linked to the upregulation of tryptophan hydroxylase 1 in the colon, an enzyme capable of limiting the rate of 5-HT production^[79,80]^. However, considering that 5-HT is unable to cross the BBB, the direct influence of changes in intestinal 5-HT levels in the brain has not yet been demonstrated^[81]^. In addition to the reduction of 5-HT, depletion of N-acetyl aspartic acid levels has been found in patients with epilepsy. This trend was also observed in the epileptic piglet model, which allowed researchers to highlight an association between the levels of *Ruminococcus* and N-acetyl aspartic acid potentially mediated by serum cortisol levels^[82]^. Neurotransmitters such as dopamine, 5-HT, acetylcholine, and norepinephrine have been found to be closely related to the onset of seizures and epilepsy due to their indirect influence on brain function mediated by the enteric nervous system, the vagus nerve, and the regulation of receptor expression at the peripheral level^[13,83]^. However, the direct involvement of the GM and mechanistic relationships underlying neurotransmitter regulation in the context of MGB axis in epilepsy have yet to be fully elucidated.

### The hypothalamic-pituitary-adrenal axis

The hypothalamic-pituitary-adrenal (HPA) axis is central to responses to stress, one of the key promoters of epilepsy induction^[84]^. This axis is involved in the secretion of corticotropin-releasing factor and the subsequent release of glucocorticoids such as cortisol, corticosterone, deoxycorticosterone, and corticotrophin. Higher levels of glucocorticoids have been found in epileptic patients, emphasizing the importance of the HPA axis in this neurological disorder. In addition to inputs from many different brain regions, including the prefrontal cortex, hippocampus, amygdala, and bed nucleus of the stria terminalis, negative feedback from glucocorticoids also plays an essential role in regulating the HPA axis ^[85]^. Chronic stress might upregulate glucocorticoids, which might enhance glutamatergic signaling and induce seizures^[86]^.

Corticotropin-releasing hormone and corticosterone may also promote seizure activities, while most deoxycorticosterones exhibit anticonvulsant properties^[87,88]^. Although accumulating evidence suggests a correlation between the HPA axis and GM, the specific mechanisms underlying this interchange have not yet been fully elucidated^[89]^. However, a central role for cortisol in influencing the MGB axis in multiple ways has been proposed. Specifically, a direct effect of cortisol on gut function has been proposed, as cortisol receptors are expressed on different types of gut cells, from epithelial and immune cells to EECs^[90-92]^. The effects induced by cortisol are also reflected in the composition and functionality of the GM, simultaneously altering intestinal transit, intestinal permeability and the bioavailability of nutrients ingested with the diet, which in turn affect key aspects of the GM layout, such as diversity^[92]^. Cortisol levels may also have a direct impact on the CNS following binding to glucocorticoid receptors, which are neuronally expressed in various brain regions, such as the hippocampus, amygdala, and prefrontal cortex. Microorganisms residing in the GIT can also activate stress circuits in the CNS via the vagus nerve and other sensory neurons of the enteric nervous system, supporting signaling between the GM and the CNS^[93]^. Finally, regulation of the HPA axis by GM may be attributable to the modulation of circulating cytokine levels, ultimately influencing hypothalamic functions^[94]^. Studies conducted on GF or specific-pathogen-free murine models have confirmed the pituitary and adrenal stress-dependent activation mediated by the GM, highlighting an alteration of gene expression responsible for regulating the hormone pathway of corticotropin release in the colon^[95]^. While intriguing, these findings need further validation to fully elucidate the intricate relationship between the HPA axis, GM, and epilepsy.

## ALTERATIONS OF THE GUT MICROBIOTA IN EPILEPSY

According to Lum *et al*., GM dysbiosis and the frequency of seizure occurrence may be two core epilepsy characteristics strictly connected with one another^[17]^. Accumulating evidence suggests that GM composition could be considered a signature/biomarker of epilepsy. For example, a recent study by Şafak *et al.* showed an increased abundance of Proteobacteria in 30 patients with idiopathic focal epilepsy compared to 10 healthy subjects^[96]^. In particular, the genera *Campylobacter*, *Delftia*, *Haemophilus*, *Lautropia*, and *Neisseria* were significantly higher in patients with epilepsy. Conversely, Firmicutes, *Bacteroidetes*, and Actinobacteria were higher in the healthy group. Another study, conducted by Xie *et al.*, compared the feces of 14 infant patients with those of 30 healthy infants, aged 2 years-3 years^[97]^. Higher GM diversity was found in healthy infants, but, in contrast to the study of Şafak *et al.*, the phylum Firmicutes was predominant in epileptic patients^[96]^. In the work of Dong *et al.*, the authors found that the relative abundances of *Fusobacteri*a and *Verrucomicrobi*a at the phylum level and *Alloprevotella* at the genus level were increased in 41 epileptic patients compared with 31 healthy controls^[98]^. In this study, several types of epilepsy were considered, including focal, general, structural, infectious immune and hereditary epilepsy. The trend of Verrucomicrobia was consistent with what was seen in a previous study^[99]^. Interestingly, *Verrucomicrobia* has been associated with the increase in glutamate and glutamine, along with the decrease in 5-HT, indicating that this taxon may be involved in the pathogenesis of epilepsy^[74]^. Additionally, at the species level, epilepsy correlated with the proinflammatory taxa *Ruminococcus gnavus*, *Fusobacterium mortiferumis*, and *Bacteroides fragilis*. In particular, the positive correlation of *Fusobacterium* with epilepsy confirmed the results observed by Şafak *et al^.^*^[96]^. Recently, Xu *et al*. analyzed the GM composition of 29 infants with severe epileptic encephalopathy, known as West syndrome (generally with onset between 3 months and 2 years of age). This condition is characterized by infantile spasms, hypsarrhythmia recorded on interictal electroencephalographs, and neurodevelopmental delay. Again, the authors found that the main difference between patients and healthy controls was the relative abundance of a *Verrucomicrobia* member, *Akkermansia*, which was overrepresented in the former^[100]^.

Regarding animal models, the study of Citaro *et al.* involved an animal model of genetic absence epilepsy (i.e., brief seizures during which the patient is unresponsive, typically seen in children aged 4 years-12 years), WAG/Rij rats, and Wistar rats as a nonepileptic control, monitoring the disease symptoms and GM profiles at 1 month, 4 months, and 8 months of age^[71]^. *Bacteroidetes* and Firmicutes were the most dominant phyla in both WAG/Rij and control rats at 1 month of age. The main differences between the two groups were found at 4 months, when a lower abundance of an unclassified genus of S24-7, and a higher abundance of *Odoribacter* and an unclassified genus of *Rikenellaceae* were detected in WAG/Rij rats^[71]^. Medel-Matus *et al.* investigated the disruption of intestinal barrier integrity due to post-traumatic epilepsy caused by brain injury in adult male Sprague-Dawly rats^[101]^. Lateral fluid percussion injury was used to induce the development of post-traumatic epilepsy symptoms, which resulted in a significant change in beta diversity in the GM. Furthermore, rats developing post-traumatic epilepsy were enriched in *Lachnospiraceae* and *Ruminococaceae*, and depleted in *Muribaculaceae*, compared to the group that received lateral fluid percussion injury but did not develop epilepsy.

### Two-way relationship between gut microbiome and antiepileptic drug resistance

When antiseizure medications are taken into account and a distinction is made between drug-sensitive and resistant epileptic patients, GM dysbiosis can become even more evident and discriminating. In a recent work by Lee *et al*., 44 adult patients with epilepsy were divided into two groups, i.e., drug-responsive and drug-resistant, defining drug-resistant epilepsy as “the failure of adequate trials of using antiepileptic drug, either as sequential monotherapies or in combination, to achieve “seizure freedom”^[102,103]^. Although no significant differences were found in GM diversity, the composition was markedly different between the two study groups. More precisely, the relative abundances of *Bacteroides finegoldii* and *Ruminococcus_g2* were higher in the drug-responsive group, whereas the drug-resistant group showed a greater relative abundance of *Negativicutes*, a bacterial genus belonging to the phylum Firmicutes^[102]^. Furthermore, patients with a normal brain magnetic resonance image showed a higher amount of *B. finegoldii*, while the genus *Shigella* and the order Veillonellales were more abundant in patients with abnormal brain imaging. In patients with a normal electroencephalogram, *Bifidobacterium* was relatively more abundant, while the genera *Klebsiella* and *Streptococcus* were relatively more represented in patients with an abnormal electroencephalogram. The study by Peng *et al.* showed increased proportions of several normally subdominant bacteria, including *Clostridium XVIII*, *Atopobium*, *Holdemania*, *Dorea*, *Saccharibacteria*, *Delftia*, *Coprobacillus*, *Paraprevotella*, *Ruminococcus*, *Gemmiger*, *Akkermansia*, *Neisseria*, *Coprococcus*, *Fusobacterium*, *Methanobrevibacter*, *Phascolarctobacterium* and *Roseburia*, in the GM of patients with drug-resistant epilepsy^[19]^. Interestingly, the GM composition of the drug-sensitive group was more similar to that of healthy controls. Furthermore, the abundance of Firmicutes was higher in the drug-resistant group, while the abundance of Bacteroidetes was higher in the drug-sensitive group. Finally, *Verrucomicrobi*a was more abundant in the drug-resistant group than in the drug-sensitive group and healthy controls. A brilliant study by Ilhan *et al.* was conducted in 2022 using the human epithelial cell line HT-29 to investigate the anti-commensal effects of commonly prescribed antiepileptic medications (e.g., valproate, carbamazepine) as well as their excipients (e.g., methyl-paraben, propyl-paraben) on core bacterial species of healthy infants and young children^[104]^. Carbamazepine, lamotrigine*,* and phenytoin inhibited at least 25% of the growth of 10, 19, and 12 strains at a concentration of 2,000 μm. Although the stationary growth phase of most strains was shortened by the majority of the active principles tested, the species *Staphylococcus caprae*, *Dorea longicatena*, *Escherichia coli*, and *Klebsiella aerogenes* showed greater growth with topiramate, carbamazepine, lacosamide, and phenytoin than with negative controls. The physicochemical properties of compounds could affect the growth of specific commensal strains: for example, hydrophobic active compounds, such as topiramate or carbamazepine, mutually reduced bacterial growth compared to hydrophilic compounds. In conclusion, the authors found that the active principles of the most common antiepileptic drugs have species-specific effects on bacterial growth, but phylogeny, including cell wall structure, together with the physicochemical properties of the active compounds, had a confounding impact on the growth response of the microorganisms^[104]^. However, it should be noted that a few years earlier, a clinical study by Watkins *et al.* on the GM and metabolome of infants showed only mild effects of antiseizure treatments with carbamazepine on selected GM bacteria^[105]^.

On the other hand, bioaccumulation by GM and GM-derived metabolism is known to have a significant impact on the bioavailability, efficacy and toxicity of a wide range of non-antibiotic drugs^[106-108]^. In addition, the GM may affect pharmacokinetics, side effects and drug response through indirect mechanisms, including modulation of host pathways involved in drug metabolism and transport, competition of microbiome-derived metabolites for the same host targets, reactivation of detoxified drug metabolites, and modulation of immune system activity and inflammatory processes^[109-113]^. For example, a recent study showed that two-thirds of 271 orally administered drugs were metabolized by at least one bacterial strain, suggesting that this process is more common than previously thought^[114]^. In this study, levels of lamotrigine, oxcarbazepine, and phenytoin were all markedly reduced by gut-derived bacteria, with *in vitro* levels of phenytoin decreasing by up to a third over the course of 12 hours of incubation. *In vivo* studies in murine models suggested that the GM contributes 78% and 66%, respectively, to the systemic levels of the two major metabolites of clonazepam^[106]^, strengthening the hypothesis of a significant contribution of GM to drug resistance phenomena. However, information on the mechanistic relationship between the GM and drug-resistant epilepsy remains limited. Identifying differences in GM composition and functionality between drug-sensitive and drug-resistant epileptic patients is a fundamental first step, but further studies in larger cohorts, using multi-omics approaches and animal models, are needed to definitively uncover the underlying mechanisms.

## GUT MICROBIOME MODULATION VIA DIETARY INTERVENTION STRATEGIES TO TACKLE EPILEPSY

Accumulating evidence suggests that non-pharmacological intervention strategies aimed at modulating the GM may constitute a potential therapeutic approach for intractable epilepsy. Restoring intestinal eubiosis could indeed reduce the onset of seizures by modulating epilepsy-related mechanisms. As discussed earlier, intestinal microorganisms may also affect the metabolism and absorption of antiepileptic drugs, in turn influencing therapeutic efficacy and responsiveness to drug treatment^[115]^. Among GM modulation intervention strategies, the administration of prebiotics, probiotics, synbiotics, and postbiotics up to fecal microbiota transplantation (FMT) constitute a valid option and have been summarized in several brilliant literature reviews^[21,116,117]^. For example, in a clinical trial of 45 patients with drug-resistant epilepsy, treatment with a probiotic mixture (*Streptococcus thermophilus*, *Lactobacillus acidophilus*, *Lactobacillus plantarum*, *Lactobacillus paracasei*, *Lactobacillus delbrueckii* subsp. *bulgaricus*, *Bifidobacterium breve*, *Bifidobacterium longum*, and *Bifidobacterium infantis*) was shown to reduce seizure frequency by 50% or more in 28.9% of individuals^[118]^. As for FMT, He *et al.* have reported the first case of a patient with Crohn’s disease and epilepsy whose symptoms were successfully controlled following healthy donor transplantation^[119]^. To our knowledge, there are no clinical trials of synbiotic and postbiotic administration in epileptic patients, while a Canadian clinical trial of prebiotic administration in a pediatric population is ongoing, but preliminary data are not yet available (NCT04705298).

On the other hand, diet is the most significant and major driver of GM composition^[120,121]^, and in the present section, we will review one particular dietary regimen and its variants, i.e., the KD, which is gaining increasing attention in the treatment of multiple types of epilepsy.

### Ketogenic diet and its relevance in epilepsy

KD is a specific type of diet capable of triggering the synthesis of ketone bodies, such as acetone and acetic acid, following the depletion of glycogen stores in the liver. While acetone is rapidly utilized, aceto-acetic acid is converted into β-hydroxybutyrate, which can be used by the cells for energy purposes. Ketone bodies are synthesized in the liver to balance the lack of glucose availability at the level of neuronal cells in the CNS. Since β-hydroxybutyrate can cross the BBB^[122]^ and directly enter the Krebs cycle, this compound constitutes a more efficient source of energy than glucose^[123]^. Numerous varieties of KD are available to date, including the traditional KD, the medium-chain triglyceride diet, the modified Atkins diet, and the low glycemic index treatment^[124]^. Classic KD was the first diet of this kind to be described one hundred years ago by Wilder in 1921. This type of diet provides for the intake of long-chain triglycerides, which represent the main portion of the lipid intake. The medium-chain triglyceride diet was introduced in the 1950s and contained mainly octanoic (C8) and decanoic (C10) fatty acids, distinguishing itself from the classic KD due to the higher yield of ketones per kilocalorie of energy^[125-127]^. In the modified Atkins diet, developed in 2003, about 65% of total caloric intake comes from fat and does not require hospitalization to be administered. This innovative aspect makes this type of diet the most self-administrated KD variant^[128]^. Finally, the low glycemic index treatment was introduced in 2005. This dietary treatment involves consuming foods with a glycemic index below 50, where 100 is the glycemic index of reference (i.e*.*, glucose)^[124,129]^. Due to its peculiarities, KD is not safe and can have various side effects, among the most frequent gastrointestinal disorders, as well as dyslipidemia that can lead to an increased risk of cardiovascular disease^[130,131]^. In particular, constipation was the most common adverse effect, followed by vomiting, diarrhea, hunger, abdominal pain, gastroesophageal reflux, and fatty diarrhea^[130]^. In terms of frequency, dyslipidemia (hyperlipidemia, hypercholesterolemia, and hypertriglyceridemia), hyperuricemia, lethargy, and infectious diseases followed gastrointestinal symptoms. Infrequent but severe adverse effects included gastritis, thrombocytopenic purpura, and respiratory failure. Sixty-six deaths not directly related to KD have been reported in several meta-analyses^[130,132-134]^. According to other studies, the most common adverse effects are anorexia, weight loss, growth problems, and metabolic diseases. Adverse effects can also be divided into short- and long-term effects. In particular, short-term adverse effects of KD include changes in lipid profiles and hypercalciuria, while long-term adverse effects include anorexia, kidney stones, growth problems, and excessive weight loss. Often, fine-tuning of the KD is sufficient to reduce adverse GIT effects. However, many of the patients on KD enrolled in the mentioned studies were infants, children, or other frail patients, who had severe disabilities and were extremely vulnerable. Monitoring for gastrointestinal symptoms, hypoglycemia and excess ketosis after initiation of KD in infants is of paramount importance^[132,133]^. Due to its restrictiveness and side effects, KD requires strict dietary and medical control^[135]^.

### Influence of the ketogenic diet on the microbiota-gut-brain-axis

As might be expected, KD has a strong impact on the GM layout, even at a high phylogenetic level, as demonstrated by numerous studies both in animal models and in humans. For example, in children with refractory epilepsy, Zhang *et al.* reported an increased relative abundance of Bacteroidetes and a decreased relative abundance of Firmicutes and Actinobacteria^[136]^. Xie *et al*. reported a dramatic decrease in Proteobacteria and a significant increase in *Bacteroides*^[97]^. According to the authors, the increase in *Bacteroides* may be closely related to the antiseizure effects of KD through the regulation of IL-6 and IL-17 secretion in dendritic cells, a process associated with seizure severity in epileptic patients. In general, KD decreases the GM biodiversity, alters the proportion of specific microbial taxa (e.g., *Akkermansia* and *Parabacteroides*) with a consequent decrease in the levels of γ-glutamyl amino acids, which are associated with seizure susceptibility^[18,137,138]^, and affects the levels of other microbially derived metabolites such as SCFAs, lactate and H_2_S^[138]^. With specific regard to *A. muciniphila*, it has also been proposed that its beneficial effects are related to changes in plasma levels of SCFAs^[138]^. However, it should be noted that a previous study showed that very low caloric KD reduced *A. muciniphila* levels^[139]^. Furthermore, *Desulfovibrio*, which is considered a harmful bacterial genus as it produces H_2_S that causes damage to the intestinal mucosal barrier, appeared reduced after KD treatment^[137,138]^. In a study of KD administration in children with severe epilepsy, Lindefeldt *et al*. found a significant depletion of *Bifidobacterium* and *Eubacterium rectale*, with an increased representation of *E. coli*^[139]^. The depletion of bifidobacteria was confirmed in the study by Ang *et al*. in nonepileptic overweight/obese patients, who also showed that the KD-associated GM reduced the levels of proinflammatory Th17 cells in the gut^[140]^. According to the authors, this immune response may underlie the efficacy of KD in the treatment of refractory epilepsy and possibly other diseases associated with increased Th17 activation. Regarding SCFAs, Gong *et al*. showed that their fecal levels increased in patients with drug-resistant epilepsy treated with KD for six months and were highly correlated with specific gut bacteria (e.g., acetate with *Lachnoclostridium*, butyrate with *Anaerostipes*, propionate with *Fusicatenibacter*, and isobutyrate and isovalerate with *Collinsella*). Interestingly, all patients had similar SCFA levels before starting KD^[141]^. Conversely, in a pilot study conducted by Ferraris *et al.* on 7 drug-resistant epileptic patients, the researchers showed that after only 1 month of KD, total SCFAs were reduced by 55%, with a reduction of 64% in acetate, 33% in propionate and 20% in butyrate^[142]^. Unfortunately, these findings are controversial and require further investigation in larger cohorts.

In summary, it is expected that changing the diet of an epileptic patient to KD will affect the composition and functionality of the GM, and thus the pool of metabolites and messengers produced, including immune responses, which in turn may affect the entire MGB axis mediating the host response to KD.

### Clinical trials focused on ketogenic diet for the treatment of epilepsy

As of July 2023, there were five registered clinical trials (ClinicalTrials.gov search terms: “gut microbiome”, “epilepsy”, and “ketogenic diet”) on KD-based therapeutic interventions in patients with neurological disorders such as epilepsy, Parkinson’s disease, and autism spectrum disorder (ASD) [Table 1]. Only two have been completed and two are still recruiting patients, while the last one has yet to start enrollment. With the exception of the latter (focused on adults with Parkinson’s disease), all other clinical trials evaluate the impact of KD on pediatric patients with epilepsy and ASD aged 6 months to 21 years.

**Table 1:** Clinical trials registered on ClinicalTrials.gov (as accessed on July 2023) focusing on the impact of the ketogenic diet in treating epilepsy and other neurological conditions, and including the gut microbiome

**Neurological condition**	**Title**	**N**	**Age**	**Status**		**Intervention**	**Location**	**NCT Number**
Epilepsy	Epigenetics and Gut Microbiota in Children With Epilepsy (EpiMICRO)	60	2-17 y	Recruiting		Ketogenic Diet.	Norway	NCT04063007
Epilepsy	Ketogenic Diet Program for Epilepsy	30	6 m-18 y	Completed		Ketogenic Diet.	United States	NCT02497105
Epilepsy (DRE)	Role of the Gut Microbiota in Pediatric Epilepsy (EPBiome)	45	2-18 y	Recruiting		Ketogenic Diet. Dietary Supplement: inulin	Canada	NCT04705298
Autism Spectrum Disorder	Ketogenic Diet Therapy for Autism Spectrum Disorder	119	2-21 y	Completed		Ketogenic Diet.	United States	NCT02477904
Parkinson’s Disease	Ketogenic Diet Interventions in Parkinson's Disease: Safeguarding the Gut Microbiome (KIM)	50	45-85 y	Not yet recruiting		Mediterranean-Ketogenic Diet. Dietary Supplement: medium-chain triglyceride oil	Canada	NCT05469997

DRE: Drug-resistant epilepsy.

As for the completed trials, a first interventional trial in Hawaii (United States) focused on 30 pediatric participants (0 year-18 years of age) with a primary diagnosis of epilepsy (NCT02497105). The children were assigned in parallel to two study groups according to whether or not they received the KD, namely a high-fat, low-carbohydrate, and moderate-protein diet (i.e., epilepsy/KD and epilepsy/non-KD). Changes from baseline in the biochemical profiles, ketone levels, and core epilepsy symptoms, i.e., seizure frequency and severity, were evaluated after three and six months on KD. Although this clinical trial has been completed, the results have not yet been published. In the second completed Hawaiian trial, Lee *et al.* assessed the effectiveness of KD in treating ASD (NCT02477904)^[143]^. The trial was conducted in a cohort of 119 children (aged 2 years-21 years) diagnosed with ASD who were assigned in parallel to three study groups based on whether or not they had ASD and received KD (i.e*.*, ASD/KD, ASD/non-KD, and non-ASD/non-KD). The clinical trial enrolled an ethnically diverse population with varied genetic background and environmental exposure. In particular, the researchers aimed to assess changes from baseline after three and six months of KD in core ASD features, blood composition, and GM. They hypothesized that participants diagnosed with ASD following KD (i.e., the ASD/KD group) would have significantly reduced ASD symptoms compared to those with ASD who did not follow KD (ASD/non-KD group). Different biochemical profiles and higher serum/urinary ketone levels are also expected in the ASD/KD group compared to the ASD/non-KD group.

In a Norwegian prospective, non-randomized study (EpiMICRO), the researchers will follow 60 children with drug-resistant epilepsy for 4 weeks before initiating KD and during the 12-week treatment. More precisely, the researchers will investigate the influence of KD on the GM, as well as the impact on epigenetics, quality of life, and eventual adverse effects (NCT04063007). However, recruitment is still ongoing.

A second American randomized clinical trial, conducted as part of the EPBiome study (NCT04705298), will focus on a cohort of 45 pediatric patients with drug-resistant epilepsy, who will be assigned in parallel to two study groups based on whether or not they receive KD with prebiotic fiber. The objectives are to evaluate the feasibility of a 12-week dietary intervention with the prebiotic oligofructose-enriched inulin (4 grams daily for ≤ 6 years; 8 grams for > 6 years). The authors speculate that prebiotic consumption will modulate GM and translate effects on seizure frequency.

In a recent Canadian clinical trial, the investigators will enroll 50 adult patients with Parkinson’s disease for two 8-week interventions in randomized order using a cross-over design, separated by a washout period of 8 weeks (NCT05469997). Specifically, the participants will adhere first to a Mediterranean diet supplemented with medium-chain triglyceride oil and second to a modified Mediterranean-KD, limiting the intake of carbohydrates (10% of total daily calories) to promote calorie intake from plant-based fats (70%-75%) and lean proteins (15%-20%). The study aims to investigate the safety of modified Mediterranean-KD interventions as alternatives to classical KD, avoiding perturbations of GM.

## CONCLUSION

The understanding of the impact of GM on health and disease is constantly deepening thanks to the techniques of analyzing multi-omics data and integrating them with pathophysiological aspects of the host. Accumulating evidence has highlighted the key role of the MGB axis in epilepsy, which exploits mechanisms involving the autonomic, enteric, neuroendocrine and neuroimmune nervous systems. In clinical practice, KD has long been used as an effective treatment for intractable epilepsy. KD has also been shown to have a major impact on GM composition and function, with cascading effects on seizure frequency and epilepsy threshold. However, there is still a lack of knowledge about the precise mechanisms of action of this dietary treatment in reducing seizures, which patients are most likely to benefit from and respond to treatment, and potential adverse effects, including long-term effects, such as anorexia and growth problems. In addition to addressing these issues, future studies should unravel all the communication pathways along the MGB axis and identify innovative diagnostic biomarkers and therapeutic targets for refractory epilepsy, which could include various actors involved in the MGB axis, such as autonomic nervous system components, metabolic and immunological pathways, and GM-related neurotransmitters and/or other neuroactive products. Once this knowledge is obtained, it will be possible to optimize KD, reducing the incidence of both short-term and long-term complications, and thus making it safer for effective treatment of different types of epilepsy. Not less importantly, such knowledge could lead to the design of other precision modulation strategies (possibly involving prebiotics, probiotics, synbiotics, postbiotics, FMT, or *ad hoc* consortia) of the MGB axis to treat this complex neurological condition.

## DECLARATIONS

### Copyright

© The Author(s) 2023.
